# Use of *Caenorhabditis elegans* as a model to study Alzheimer’s disease and other neurodegenerative diseases

**DOI:** 10.3389/fgene.2014.00279

**Published:** 2014-09-05

**Authors:** Adanna G. Alexander, Vanessa Marfil, Chris Li

**Affiliations:** ^1^Department of Biology, City College of New YorkNew York, NY, USA; ^2^Department of Biology, The Graduate Center, City University of New YorkNew York, NY, USA

**Keywords:** *apl-1*, *C. elegans*, Alzheimer’s disease, ALS, Parkinson disease, model systems

## Abstract

Advances in research and technology has increased our quality of life, allowed us to combat diseases, and achieve increased longevity. Unfortunately, increased longevity is accompanied by a rise in the incidences of age-related diseases such as Alzheimer’s disease (AD). AD is the sixth leading cause of death, and one of the leading causes of dementia amongst the aged population in the USA. It is a progressive neurodegenerative disorder, characterized by the prevalence of extracellular Aβ plaques and intracellular neurofibrillary tangles, derived from the proteolysis of the amyloid precursor protein (APP) and the hyperphosphorylation of microtubule-associated protein tau, respectively. Despite years of extensive research, the molecular mechanisms that underlie the pathology of AD remain unclear. Model organisms, such as the nematode, *Caenorhabditis elegans*, present a complementary approach to addressing these questions. *C. elegans* has many advantages as a model system to study AD and other neurodegenerative diseases. Like their mammalian counterparts, they have complex biochemical pathways, most of which are conserved. Genes in which mutations are correlated with AD have counterparts in *C. elegans*, including an *APP*-related gene, *apl-1*, a tau homolog, *ptl-1*, and presenilin homologs, such as *sel-12* and *hop-1*. Since the neuronal connectivity in *C. elegans* has already been established, *C. elegans* is also advantageous in modeling learning and memory impairments seen during AD. This article addresses the insights *C. elegans* provide in studying AD and other neurodegenerative diseases. Additionally, we explore the advantages and drawbacks associated with using this model.

## INTRODUCTION TO ALZHEIMER’S DISEASE

Alzheimer’s disease (AD) is the 6th leading cause of death in the US and affects more than 35 million people worldwide (Alzheimer’s Disease International, 2014). AD is a neurodegenerative disease characterized by a progressive loss of memory. Most cases of AD occur sporadically in aged people (>60 years, late-onset AD) without a clear inheritance pattern. However, in 5% of the cases (familial or early onset AD) AD symptoms appear earlier and are linked with gene mutations. Both forms of AD have two main neuropathologic features: the presence of extra-neuronal amyloid plaques, often referred to as senile plaques, and intraneuronal neurofibrillary tangles (Kidd, 1964; Luse and Smith, 1964; Terry et al., 1964; Krigman et al., 1965). Amyloid plaques are aggregates of the beta-amyloid peptide (Aβ), a cleavage product of the amyloid precursor protein (APP; Glenner and Wong, 1984; Masters et al., 1985; Kang et al., 1987). Hyperphosphorylation of the microtubule associated protein tau causes its polymerization into paired helical filaments (PHFs) and, presumably, its formation into neurofibrillary tangles (Goedert et al., 1989a).

Mutations in the *APP* gene and/or the enzymes involved in APP processing (γ-secretase components presenilins, PSEN1 and PSEN2; Chartier-Harlin et al., 1991; Goate et al., 1991; Murrell et al., 1991; Levy-Lahad et al., 1995; Rogaev et al., 1995; Sherrington et al., 1995) are correlated with early onset AD. These mutations increase the levels of toxic Aβ species and promote neurodegeneration. By contrast, a recently identified mutation in *APP* affects cleavage of APP, causing less Aβ production and conferring neuroprotective benefits (Jonsson et al., 2012). Despite the significant advances made using *APP* transgenic and knockout models in mammals, unraveling the cellular role of APP has been difficult. Alternative animal models provide complementary approaches to dissecting the function of APP and tau. In this review, we discuss the latest uses of the nematode *Caenorhabditis elegans* as a model system for the study of AD. We also include a brief review of a few representative examples of how *C. elegans* is being utilized to model other neurodegenerative diseases.

### *C. elegans* AS A MODEL FOR ALZHEIMER’S DISEASE

*Caenorhabditis elegans* is a free-living, non-parasitic nematode that was first introduced as a model organism by Sydney Brenner in 1963 (Brenner, 1974). It is a small (1 mm in length), transparent roundworm, which makes it easy for manipulation, and has a short life cycle of 3 days from egg to adult at 25°C (Brenner, 1974). Under suitable growing conditions, hatched animals develop through four larval stages (L1–L4), each punctuated by a molt, to arise as an adult hermaphrodite with 959 somatic cells (Sulston and Horvitz, 1977). Its life span is between 2 and 3 weeks, which facilitates the study of its biology. Completion of the *C. elegans* genome sequence in 1998 (*C. elegans* Sequencing Consortium, 1998) demonstrated that roughly 38% of worm genes have a human ortholog, such as *APP* and *tau* (Shaye and Greenwald, 2011). Hence, *C. elegans* has many excellent advantages as an *in vivo* model for the study of AD and other neurodegenerative diseases.

## MOLECULAR PATHWAYS OF APP. SIMILARITIES AND DIFFERENCES BETWEEN MAMMALS AND *C. elegans*

### FUNCTION AND PROCESSING OF APP: NON-AMYLOIDOGENIC AND AMYLOIDOGENIC Aβ PATHWAY

**FIGURE 1:**
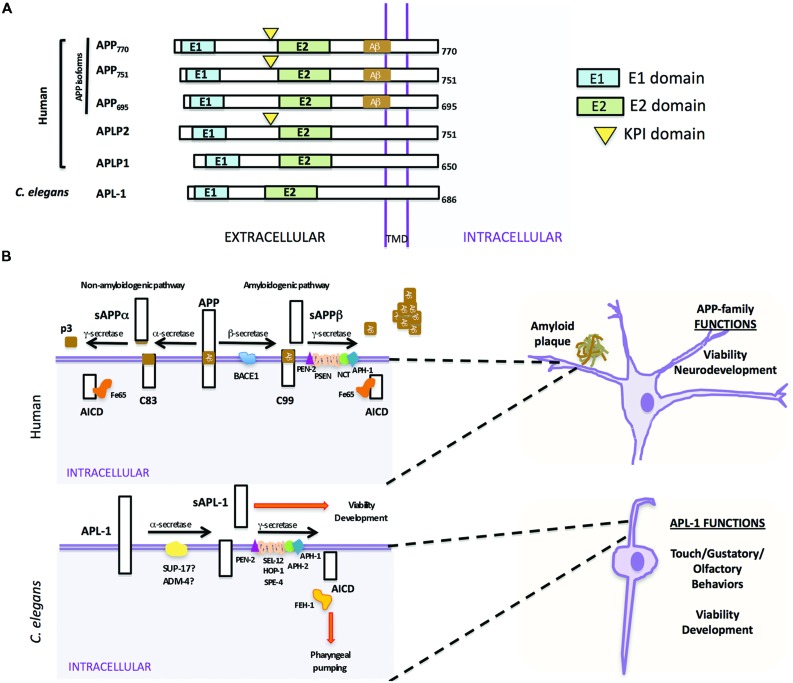
**Similarities and differences between human APP and *Caenorhabditis elegans* APL-1. (A)** Schematic representation of human APP isoforms, other members of the APP family (APLP1 and APLP2), and *C. elegans* APL-1. **(B)** Comparison between human APP proteolytic pathways (top) and *C. elegans* APL-1 proteolytic pathway (bottom). *Top*. APP can be cleaved by two different pathways. In the anti-amyloidogenic pathway, APP is first cleaved by the α-secretase to release an extracellular fragment sAPPα. The remaining APP fragment (APP-CTFα or C83) is then cleaved by the γ-secretase complex to release p3 extracellularly and the APP intracellular domain (AICD) to the cytosol. In the amyloidogenic pathway, β-secretase first cleaves APP, releasing the sAPPβ fragment. The APP-CTFβ (C99) fragment is subsequently cleaved by the γ-secretase complex, liberating the AICD to the cytosol and Aβ to the lumen. Aβ will aggregate to form amyloid plaques. *Bottom*. In *C. elegans*, APL-1 is first cleaved by the α-secretase homologs SUP-17/ADM-4, liberating the extracellular sAPL-1 that is known to regulate worm viability and development. The γ-secretase complex will then cleave the remaining APL-1-CTF to release AICD into the cytosol. General functions of the APP family and APL-1 are indicated.

The APP family of proteins contains three members, APP, APLP1, and APLP2 (Wasco et al., 1992, 1993; Sprecher et al., 1993; Sandbrink et al., 1994; Slunt et al., 1994), which are characterized by a large extracellular region containing conserved E1 and E2 domains, a single transmembrane domain, and a small cytosolic domain (Kang et al., 1987). APLP1 and APLP2 do not contain the Aβ sequence and, hence, do not produce Aβ (**Figure 1A**; Wasco et al., 1992, 1993). The APP gene family is required for viability and brain development. *APP* mouse knockouts were viable, but had behavioral and cognitive defects (Ring et al., 2007). While knockout of APLP1 resulted in postnatal growth defects (Heber et al., 2000), mice in which APLP2 was inactivated appeared wild type (von Koch et al., 1997). Double knockouts of APLP2 and either APP or APLP1, however, resulted in postnatal lethality (von Koch et al., 1997; Heber et al., 2000); the lethality of *APP/APLP2* double knockouts could be rescued by knock-in of an APP extracellular fragment, sAPPα (Weyer et al., 2011), suggesting that sAPPα is sufficient for viability. The triple knockout caused lethality and a type II lissencephaly and cortical disorganization (Herms et al., 2004). Collectively, these results suggest that APP family members have essential and redundant functions during development, including proper brain development, and these functions do not require Aβ.

In mammals, APP is processed through two proteolytic pathways, only one of which produces Aβ (**Figure 1B**; Haass et al., 1992, 1994a,b). In the non-amyloidogenic pathway, APP is first cleaved by an α-secretase within the Aβ sequence to release an extracellular fragment, sAPPα (**Figure 1B**). The remaining APP fragment (known as APP-CTFα or C83) is then cleaved by the γ-secretase complex to release the APP intracellular domain (AICD) to the cytosol. By contrast, in the amyloidogenic pathway, after cleavage by the β-secretase (BACE) to release sAPPβ, the remaining APP fragment (known as APP-CTFβ or C99) is then cleaved by the γ-secretase complex, liberating Aβ to the lumen and AICD to the cytosol (Gu et al., 2001; Sastre et al., 2001; Weidemann et al., 2002). This latter pathway is likely favored in AD patients.

Mammalian γ-secretase is a protease complex consisting of several components: presenilins 1 and 2 (PSEN1 and PSEN2), nicastrin (NCT), anterior pharynx defective (APH-1), and the presenilin enhancer (PEN-2; Yu et al., 2000; Francis et al., 2002). PSEN1 and PSEN2 are the catalytic components of the γ-secretase complex. NCT works as a stabilizing cofactor required for γ-secretase complex assembly and trafficking (Li et al., 2003; Zhang et al., 2005) and PEN-2 and APH-1 have a role in the maturation process of PSEN1 and PSEN2 (Luo et al., 2003). Besides APP, the γ-secretase complex is also involved in the proteolysis of Notch receptors, and the first identification of any PEN-2 or APH-1 ortholog was in *C. elegans* as the result of a genetic screen for modifiers of the Notch pathway (Francis et al., 2002; Goutte et al., 2002). Within the γ-secretase complex, only mutations in PSEN1 and PSEN2 have been associated with early onset AD (Levy-Lahad et al., 1995; Rogaev et al., 1995; Sherrington et al., 1995).

### PROCESSING OF *C. elegans* APL-1

In *C. elegans* there is only one APP-related gene, *apl-1*. Like human APP (Kang et al., 1987), APL-1 contains a large extracellular region whose conserved E1 and E2 domains share 46 and 49% sequence similarity to human APP, respectively, a transmembrane domain, and a relatively small cytosolic domain, which shares 71% sequence similarity to human APP (**Figure 1A**; Daigle and Li, 1993). Notably, unlike APP but similar to APLP1 and APLP2, APL-1 does not contain the Aβ sequence (Daigle and Li, 1993).

Two α-secretase proteins are present in *C. elegans*, SUP-17 and ADM-4 (Jarriault and Greenwald, 2005). They work redundantly in the cleavage of the *C. elegans* Notch homologs, LIN-12 and GLP-1 (Jarriault and Greenwald, 2005). However, no experiments thus far have tested whether SUP-17 or ADM-4 cleaves APL-1. No BACE ortholog has been identified by bioinformatic searches and no β-secretase activity that cleaves human APP has been detected in *C. elegans*, suggesting that APL-1 is only processed by the α/γ-secretase processing pathway (Link, 2006). α-secretase cleavage of APL-1 releases the extracellular fragment, sAPL-1; subsequent cleavage of APL-1-CTFα by the γ-secretase complex liberates the intracellular domain (AICD; **Figure 1B**).

The initial characterizations of human PSEN1 (then called S182) and PSEN2 (first named E5-1) described them as novel proteins with multiple transmembrane domains (Rogaev et al., 1995; Sherrington et al., 1995). The cellular functions of the presenilins were determined by their homology to the *C. elegans* protein, SEL-12 (Levitan and Greenwald, 1995). The two *C. elegans* Notch genes, *lin-12* and *glp-1*, are involved in many cell fate decisions during development, including vulval cell specification and germline development (Greenwald et al., 1983; Lambie and Kimble, 1991; Newman et al., 1995; Levitan et al., 1996) *sel-12*/PSEN was identified in a genetic screen to isolate suppressors of a dominant Lin-12/Notch multivulva phenotype (Levitan and Greenwald, 1995). Loss of *sel-12*/PSEN suppressed the Lin-12/Notch multivulva phenotype and produced a defect in egg laying that was rescued by introducing human PSEN1 or PSEN2, suggesting a conserved function between human and *C. elegans* presenilins (Levitan and Greenwald, 1995; Levitan et al., 1996). Like human PSENs (Thinakaran et al., 1996), SEL-12/PSEN is cleaved to attain its final topology (Li and Greenwald, 1996). A *C. elegans* PSEN gene family was identified and includes *sel-12*, *hop-1*, and *spe-4* (L’Hernault and Arduengo, 1992; Levitan and Greenwald, 1995; Li and Greenwald, 1997; Westlund et al., 1999); *spe-4* is exclusively expressed in male gonadal cells and will not be further discussed (Arduengo et al., 1998). Knockdown of *hop-1/*PSEN in *sel-12/*PSEN mutants showed maternal effect lethality, germline defects, and missing anterior pharynx, defects associated with loss of *glp-1*/Notch function, suggesting that *sel-12* and *hop-1* function redundantly in the LIN-12 and GLP-1/Notch pathways (Westlund et al., 1999). Similarly, mice carrying a null mutation in *PSEN1* showed embryonic lethality, skeletal defects, and disrupted somite boundaries (Shen et al., 1997), similar to the phenotypes seen in Notch1 knockouts (Krebs et al., 2000, 2003, 2004; Duarte et al., 2004; Gale et al., 2004).

In screening for novel mutants showing the *glp-1/*Notch phenotype of defective anterior pharynx, Goutte et al. (2000, 2002) identified two genes, *aph-1* and *aph-2*, which encodes the *C. elegans* NCT ortholog. Independently, Francis et al. (2002) screened for enhancers of *sel-12/*PSEN activity and identified *pen-2*. *aph-2*/NCT, *pen-2*, and *aph-1* are all required for proper Notch signaling. Human PSEN, NCT, Aph1α2, and PEN-2 were subsequently shown to physically associate and cooperatively regulate the maturation of individual components to form a proteolytically active γ-secretase complex (Kimberly et al., 2003).

### FUNCTION AND REGULATION OF APL-1

Like the mammalian APP family (Slunt et al., 1994; Lorent et al., 1995; Thinakaran et al., 1995), *apl-1* is expressed in multiple tissue types. *apl-1* expression is observed in neurons, supporting cells, and head muscles throughout development, while expression in vulval muscles, vulval cells, and hypodermal seam cells is not detected until the L4 stage to adult (Hornsten et al., 2007; Niwa et al., 2008).

Inactivation of *apl-1*, such as with the *yn10* null allele, results in a completely penetrant lethality during the first to second larval (L1–L2) transition due to a molting defect (Hornsten et al., 2007). *apl-1* activity is also necessary for later larval transitions, as RNAi knockdown of *apl-1* in an RNAi-sensitized background showed animals with molting defects during the L3–L4 and L4 to adult transitions (Wiese et al., 2010). This lethality was rescued by microinjection of an *apl-1* genomic fragment or cDNA. Hence, similar to the mammalian APP family, *apl-1* has an essential function. High levels of *apl-1* expression caused an incompletely penetrant L1 lethality (70% lethality), shortened body length, and morphogenetic, reproductive, and locomotory defects (Hornsten et al., 2007; Ewald et al., 2012b). These results indicate that levels of APL-1 must be tightly regulated as loss of APL-1 as well as high levels of APL-1 result in lethality. When *sel-12*/PSEN activity was reduced in transgenic animals with APL-1 overexpression, the 70% lethality was partially rescued, suggesting that SEL-12/PSEN regulates APL-1 cleavage and/or trafficking (Hornsten et al., 2007). The underlying basis of the loss- and gain-of-function *apl-1* lethality is still unclear, but is not dependent on activation of CED-3/ caspase or necrotic cell death pathways (Hornsten et al., 2007). Characterization of *apl-1* function may provide insights into the general function and pathways of APP, of which much is still unknown.

The *apl-1(yn5)* mutant, which contains a deletion of the region encoding the APL-1 transmembrane and cytosolic domains, produces only the extracellular domain of APL-1 (APL-1EXT) and is viable. Because APL-1EXT is not further cleaved by α-secretase, APL-1EXT is slightly larger than sAPL-1 and is expressed at high levels in *apl-1(yn5)* mutants (Hornsten et al., 2007). Hence, the APL-1 extracellular domain is sufficient for viability, similar to the rescue of APP/APLP2 double mutants by the knock-in of sAPPα (Weyer et al., 2011). However, although *apl-1(yn5)* mutants are viable, they display several phenotypes, including a slower developmental progression, decreased body length, reproductive defects, and temperature-sensitive lethality (Hornsten et al., 2007; Ewald et al., 2012b). Because these defects can be phenocopied by microinjection of APL-1EXT transgenes into wild-type animals, the phenotypes are due to overexpression of APL-1EXT and not due to loss of APL-1 signaling through its cytoplasmic domain (Ewald et al., 2012b). Interestingly, pan-neuronal expression of APL-1EXT, but not expression from muscle or hypodermal cells, is sufficient to rescue the lethality observed in *apl-1* null mutants (Hornsten et al., 2007), suggesting that the cells (i.e., neurons) from which sAPL-1 is released as well as the extracellular milieu in which sAPL-1 travels is functionally relevant. We suggest that high levels of sAPP may also contribute to the pathology seen in AD patients. Down’s syndrome patients, whose chromosome 21 trisomy includes trisomy of APP, display a high incidence of AD and intellectual disability (Zigman, 2013), perhaps contributed in small part by the high levels of APP expression.

Decreasing *apl-1* activity by RNAi resulted in hypersensitivity to aldicarb, an acetylcholinesterase inhibitor (Wiese et al., 2010). Using *apl-1* knockouts to test different *apl-1* deletion constructs, Wiese et al. (2010) determined that lack of sAPL-1 is responsible for the aldicarb hypersensitivity. These findings are consistent with mammalian studies, which show that a lack of APP and APLP2 impairs synaptic function at cholinergic neuromuscular junctions (Wang et al., 2005).

Heterochronic genes, whose differential spatiotemporal expression ensures proper progression through larval stages and transition into adulthood (Chalfie et al., 1981; Ambros and Horvitz, 1984), regulate expression of *apl-1* in hypodermal seam cells (Niwa et al., 2008). Loss of *let-7* microRNA (miRNA) function caused precocious seam cell development and vulval bursting at the adult stage, leading to death (Reinhart et al., 2000). These *let-7* phenotypes can be rescued by knockdown of *apl-1* (Niwa et al., 2008). *apl-1*, however, is not a direct target of *let-7* miRNA. NHR-25/Ftz-F1, which is a nuclear hormone receptor (NHR) that is required for completion of larval molts (Asahina et al., 2000; Gissendanner and Sluder, 2000), binds an enhancer element in the promoter of *apl-1* to regulate *apl-1* expression in seam cells (Niwa and Hada, 2010). *nhr-25*/Ftz-F1 transcripts are possible targets of the *let-7* family of miRNAs for downregulation (Hayes et al., 2006). Regulation of continued *apl-1* expression in adult seam cells and other cell types is unknown.

#### Pathways through which apl-1 functions

The *apl-1(yn5)* phenotypes require activity of the DAF-16/FOXO transcription factor, which is negatively regulated by the insulin pathway. *C. elegans* has only one insulin/IGF-1 receptor, DAF-2 (Kimura et al., 1997). Under favorable environmental conditions, such as when adequate food is present, signaling through the insulin pathway activates a conserved PI 3-kinase/AKT cascade (Morris et al., 1996; Kimura et al., 1997; Paradis and Ruvkun, 1998; Paradis et al., 1999), which causes phosphorylation of DAF-16/FOXO, thereby allowing reproductive development (Larsen et al., 1995; Gems et al., 1998; Henderson and Johnson, 2001). Phosphorylation of DAF-16/FOXO causes its sequestration in the cytoplasm (Lin et al., 2001), thereby preventing it from entering the nucleus to activate its target genes (Henderson and Johnson, 2001; Lee et al., 2001), which regulate longevity (Lin et al., 1997; Ogg et al., 1997), stress resistance (McElwee et al., 2003, 2004; Murphy et al., 2003), and dauer formation (Riddle et al., 1981; Vowels and Thomas, 1992). Environmental conditions also affect other metabolic functions, such as reproductive behavior, which is inhibited under starvation conditions (Seidel and Kimble, 2011), and body size. Starvation survival behavior is regulated by DAF-16/FOXO activity (Lee and Ashrafi, 2008) and the insulin (So et al., 2011) and DAF-7/TGFβ (Savage-Dunn et al., 2003) pathways work in parallel to regulate body length via *daf-16*/FOXO activity.

**FIGURE 2:**
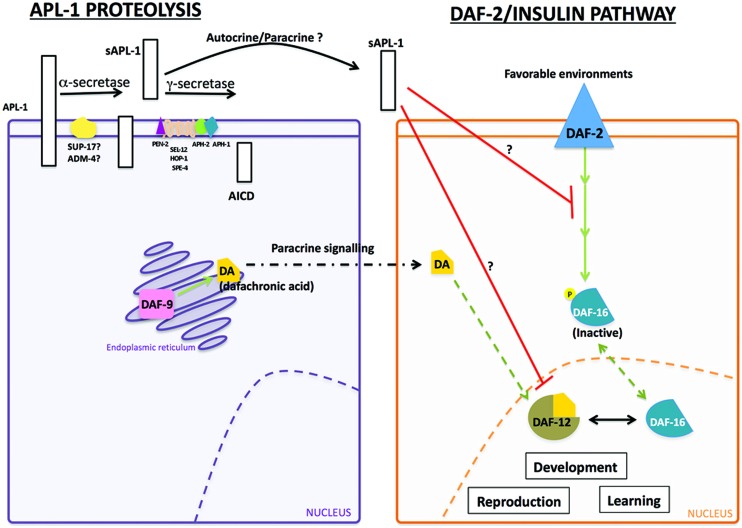
**Interaction between sAPL-1 and DAF-2 insulin/IGF-1 receptor and DAF-12/NHR pathways.** Schematic representation of APL-1 proteolytic pathway and how sAPL-1 may modulate DAF-2 insulin/IGF-1 receptor and DAF-12/NHR signaling pathways. APL-1 is cleaved by the α/γ-secretase pathway in *C. elegans*. Released sAPL-1 could act as a signaling molecule in the same cell (autocrine regulation) or in neighboring cells (paracrine regulation) to inhibit *daf-2* insulin/IGF-1 receptor and *daf-12*/NHR pathways to affect worm viability and development. The exact mechanism by which sAPL-1inhibits *daf-2* insulin/IGF-1 receptor and *daf-12*/NHR is still unknown and labeled as a question mark (see Ewald et al., 2012b).

The slowed development, decreased body size, and decreased reproductive rates of *apl-1(yn5)* mutants are dependent on *daf-16*/FOXO activity. At 20°C, mutants with decreased insulin signaling or *apl-1(yn5)* mutants showed a delayed developmental progression and shorter body length, which were enhanced when insulin signaling was decreased in *apl-1(yn5)* mutants [i.e., *daf-2(e1370); apl-1(yn5)* double mutants]; at 25°C, the *apl-1(yn5)* mutants with decreased insulin signaling went into L1 arrest (Ewald et al., 2012b). By contrast, when *daf-16*/FOXO activity was removed from *apl-1(yn5)* mutants, the delayed developmental progression, decreased reproductive rate, and smaller body length of *apl-1(yn5)* single mutants were suppressed. Furthermore, loss of *daf-16*/FOXO activity in *apl-1(yn5)* mutants with decreased insulin signaling rescued the short body length and L1 arrest phenotypes (Ewald et al., 2012b). These results suggest that sAPL-1 signals in a parallel pathway to the insulin pathway or modulates the DAF-2/insulin/IGF-1 pathway to activate *daf-16/*FOXO activity to affect developmental progression, reproductive rates, and body length (**Figure 2**). Mammalian sAPP may have similar roles in development.

Activity of the *daf-12*/NHR signals in multiple pathways to integrate environmental stimuli with metabolic needs and can modulate the insulin pathway as well as function in an independent pathway (Gerisch et al., 2001; Dumas et al., 2010). Decreasing *daf-12*/NHR activity in *apl-1(yn5)* mutants rescued the slow development, low reproductive rate, and decreased body length phenotypes (Ewald et al., 2012b). Hence, decreased insulin signaling and signaling through a parallel *daf-12*/NHR pathway converge to activate *daf-16/*FOXO for the phenotypes seen in *apl-1(yn5)* mutants. Noteworthy, levels of insulin/IGF-1 receptors are decreased in AD brains (Steen et al., 2005), and APP processing and Aβ production *in vitro* was modulated by insulin signaling (Gasparini et al., 2001). Analogous to *C. elegans*, sAPP may also act to modulate the insulin pathway.

#### Pan-neuronal APL-1 expression affects learning

In transgenic mice expressing human or mouse APP, animals showed an increased lethality and learning defects that were not correlated with Aβ deposition (Hsiao et al., 1995); similarly, doubly transgenic mice carrying transgenes with APP and PSEN1 mutations showed learning defects that were not correlated with the number of Aβ plaques (Holcomb et al., 1999). The mechanisms underlying these defects are unclear. Use of the *C. elegans* model could be informative. *C. elegans* has many sensory modalities, including smell and taste. They respond to volatile and water soluble chemicals by moving toward or away from chemoattractive or chemorepulsive stimuli, respectively. Many chemoattractants and chemorepellants have been identified and the neural circuits mediating the chemosensory response identified (Bargmann and Horvitz, 1991). For instance, when *C. elegans* is given the choice between a neutral compound and a chemoattractant, such as benzaldehyde, animals will move toward benzaldehyde; this response is mediated by the AWC neurons (Bargmann et al., 1993); similarly, ASEL, a gustatory neuron, mediates chemoattraction to sodium acetate (Bargmann and Horvitz, 1991; Pierce-Shimomura et al., 2001).

Although *apl-1* is not expressed in AWC neurons and the morphology of sensory neurons appears wild type with GFP markers, the overall chemoattractive response to benzaldyhyde and sodium acetate was decreased in *apl-1(yn10/+)* heterozygotes and transgenic animals that overexpress APL-1 [e.g., *ynIs79* (P*apl-1::apl-1::GFP*)] (Ewald et al., 2012a). The chemotaxis response was restored in APL-1 overexpression lines [e.g., *ynIs79* (P*apl-1::apl-1::GFP*)] when insulin signaling was decreased, but not when *daf-16*/FOXO activity was decreased, suggesting that *daf-16*/FOXO activity is needed for normal chemotaxis in transgenic lines overexpressing APL-1 (**Figure 2**). Pan-neuronal expression of APL-1 or targeted overexpression of APL-1 in the AWC or ASEL neurons resulted in wild-type chemotaxis responses (Ewald et al., 2012a). By contrast, ectopic expression of *apl-1* with the *snb-1* promoter, which drives pan-neuronal and multi-cell type expression, resulted in no chemotaxis response to benzaldehyde or sodium acetate (Ewald et al., 2012a). When signaling through the DAF-2/insulin/IGF-1 receptor, DAF-12/NHR, or DAF-7/TGFβ was decreased, the chemotaxis response toward benzaldehyde and sodium acetate in these transgenic lines was restored, indicating that the loss of the chemotaxis response due to ectopic *apl-1* signaling in cells outside the nervous system is dependent on insulin and TGFβ signaling.

In addition to chemosensory responses, *C. elegans* is also capable of associative chemosensory plasticity (Wen et al., 1997). For example, when benzaldehyde was paired with starvation for as short as 30 min, *C. elegans* showed a significant reduction in preference for benzaldehyde; persistence of this plasticity was positively correlated with the length of pairing time (Colbert and Bargmann, 1995; Tomioka et al., 2006; Lin et al., 2010), suggestive of stable memory formation. Both chemotaxis and associative plasticity are dependent on insulin signaling (Tomioka et al., 2006; Lin et al., 2010). Little associative plasticity, however, was observed after pairing benzaldehyde with starvation for 60 min in animals with pan-neuronal APL-1 expression (Ewald et al., 2012a). The plasticity could be restored when *daf-16*/FOXO, *daf-12/*NHR, or *daf-7*/TGFβ activity was decreased, indicating that the impaired associative plasticity with pan-neuronal APL-1 expression requires *daf-16/*FOXO, *daf-12/*NHR, and *daf-7/*TGFβ activity (Ewald et al., 2012a).

Touch habituation is another sensory characteristic affected by pan-neuronal APL-1 expression. When a gentle touch is applied to the head of the animal, *C. elegans* responds by moving backward; conversely, when touched on the tail, the animal moves forward (Chalfie and Sulston, 1981). This response to gentle body touch is mediated by six mechanosensory touch neurons (Chalfie et al., 1985). After six cycles of alternating head/tail touches, wild-type animals habituated and became unresponsive (Ewald et al., 2012a). *apl-1(yn10*/+*)* heterozygotes or transgenic animals that overexpress APL-1 showed touch habituation. By contrast, animals with pan-neuronal APL-1 expression were slow to habituate and required more alternating head/tail touch cycles before becoming habituated (Ewald et al., 2012a). Collectively, these results indicate that pan-neuronal overexpression of APL-1 causes learning defects. These results parallel those seen in mammalian models in which overexpression of APP leads to cognitive defects, independent of Aβ aggregates (Hsiao et al., 1995; Simón et al., 2009), thereby suggesting that sAPP activity, in addition to Aβ aggregates, contributes to cognitive defects. Whether these cognitive defects depend on the DAF-16/FOXO transcription factor and/or TGFβ signaling remains to be tested.

### APL-1 TRAFFICKING IS IMPORTANT FOR SYNAPTIC TRANSMISSION

APL-1, like APP (Koo et al., 1990), is transported from the cell body to synapses (Wiese et al., 2010). UNC-108, which is a neuronally expressed GTPase and localizes to the Golgi complex and early endosomes (Mangahas et al., 2008; Edwards et al., 2009), is involved in the maturation of dense core vesicles (Borgonovo et al., 2006; Mangahas et al., 2008; Edwards et al., 2009; Sumakovic et al., 2009) and the packaging of APL-1 into mature dense core vesicles (Wiese et al., 2010). Both UNC-116/kinesin-1 and UNC-104/KIF1A/kinesin-3 are involved in the anterograde transport of APL-1 (Wiese et al., 2010; Arimoto et al., 2011), but only UNC-116/kinesin-1 and dynein motors are responsible for the retrograde transport of APL-1 back to the cell body (Arimoto et al., 2011). The rates of anterograde and retrograde transport of APL-1 vesicles are 1.1 μm/s and 1.6 μm/s, respectively (Arimoto et al., 2011). Hence, APL-1 is transported similarly as in mammalian models where kinesin-1 is responsible for APP axonal transport (Koo et al., 1990). Surprisingly, mutations in *unc-116*/kinesin-1 and *unc-104/*KIF1A/kinesin-3 both caused decreased levels of APL-1 expression without affecting transcript levels, suggesting that without transport motors, APL-1 does not accumulate in cell bodies because of protein degradation (Wiese et al., 2010; Arimoto et al., 2011). APL-1 is also internalized from the cell surface of neurons via a RAB-5-dependent endocytosis (Wiese et al., 2010).

### AICD INTRACELLULAR TRAFFICKING

The Fe65 family of proteins binds the cytoplasmic YENPTY sequence of APP, APLP1, and APLP2, via their PTB2 domain (Guénette et al., 1996; Zambrano et al., 1997; Duilio et al., 1998; Russo et al., 1998). Likewise, the sole family member ortholog in *C. elegans*, FEH-1, has a WW domain and PTB1 and PTB2 domains, which closely resemble those of the Fe65 family, and the PTB2 domain of FEH-1 interacts with APL-1 (Zambrano et al., 2002).

FEH-1 is expressed in pharyngeal muscle and neuronal processes and is necessary for survival. Inactivation of *feh-1* caused an incompletely penetrant embryonic lethality. Survivors showed little pharyngeal pumping and were unable to feed, thereby resulting in L1 arrest (Zambrano et al., 2002). Decreasing *feh-1* activity or decreasing *feh-1* dosage caused pharyngeal pumping rates to increase, suggesting that the rate of pharyngeal pumping is *feh-1* dosage dependent. However, the functional significance of FEH-1 and APL-1 interactions is unclear as *apl-1(yn5)* mutants, which do not have an AICD domain, and *apl-1(yn10/+)* heterozygotes do not have defective pumping rates (Ewald et al., 2012b).

### INVESTIGATING THE AMYLOID HYPOTHESIS OF AD IN *C. elegans*

Aβ peptide, the cleavage product of APP believed to underlie the pathology of AD (Glenner and Wong, 1984; Masters et al., 1985; Gorevic et al., 1986; Selkoe et al., 1986), is not present in APL-1 (Daigle and Li, 1993) nor does *C. elegans* possess β-secretase activity to produce Aβ (Link, 2006). Nevertheless, *C. elegans* provides a powerful *in vivo* genetic system to study the effects of neurotoxic Aβ through transgene analysis (Shankar et al., 2008). Many transgenic strains have been generated in which a signal sequence followed by the human Aβ sequence is expressed in all cells, in all neurons, in specific subsets of neurons, or in muscle cells (**Figure 4**). These strains produce either Aβ_1-42_ or Aβ_3-42_.

AD is a late onset neurodegenerative disease. *C. elegans* expressing human Aβ_3-42_ in muscle tissue (Link, 1995; Link et al., 2001) showed an age-dependent paralysis at 20°C (Cohen et al., 2006; McColl et al., 2009); paralysis occurred more rapidly and more severely when Aβ_1-42_ was produced at 25°C (McColl et al., 2009). The level of muscle paralysis was significantly decreased when insulin signaling was decreased (Cohen et al., 2006). Furthermore, inhibiting *daf-16*/FOXO and *hsf-1,* which encodes a heat shock protein transcription factor (Hsu et al., 2003; Morley and Morimoto, 2004), reversed the effects of decreased insulin signaling (Cohen et al., 2006). Hence, the paralysis effects of Aβ correlates with age and is dependent on insulin signaling.

Since aggregated Aβ is toxic to neurons (Glenner and Wong, 1984; Masters et al., 1985; Gorevic et al., 1986; Selkoe et al., 1986) and causes muscle paralysis in *C. elegans*, molecules and pathways that can prevent the formation or promote the disassembly of Aβ aggregates can be screened for in *C. elegans*. For instance, when *C. elegans* extracts are incubated with aggregated human Aβ_3-42_ in the presence or absence of protease inhibitors, disaggregation occurred, but disaggregation did not occur when extracts were either heated to denature proteins or incubated with proteinases (Bieschke et al., 2009). Hence, an unidentified protein or protein complex in *C. elegans* extracts can disaggregate Aβ_3-42_ aggregates.

Several orthologs to human heat shock (HSP) chaperone proteins were found to interact directly with Aβ_3-42_ in *C. elegans*. *C. elegans* HSP-16 proteins, HSP-16-1, HSP-16-2, and HSP-16–48, orthologs of αB-crystallin, bound intracellular Aβ_3-42_ monomers and soluble Aβ_3-42_ oligomers, but not fibrillar Aβ_3-42_ (Fonte et al., 2002). Moreover, *hsp-16* transcript levels were upregulated in Aβ_3-42_ transgenic lines, but whether these chaperone proteins protect against or promote Aβ paralysis is unclear (Fonte et al., 2002). By contrast, increased expression of the HSP70 chaperones had a protective role by suppressing paralysis (Fonte et al., 2008). These results are consistent with human studies showing that HSP70 and αB-crystallin were upregulated in AD brains (Hamos et al., 1991; Perez et al., 1991; Shinohara et al., 1993; Renkawek et al., 1994; Yoo et al., 2001) and binds Aβ (Liang, 2000).

Transgenic lines in which Aβ is expressed in glutamatergic neurons showed age-dependent neurodegeneration, whereby 7-day adults showed 75% glutamatergic neurodegeneration (Treusch et al., 2011). This degeneration was suppressed when genes involved in clathrin-mediated endocytosis, such as *unc-11*, *unc-26*, *Y44E3A.4*, *C. elegans RTS1* ortholog, *C. elegans ADE12* ortholog, and human *CRMI*, were co-expressed with Aβ, and enhanced when a *PBS2/MAP2K4* mitogen-activated protein kinase transgene was co-expressed with Aβ in glutamatergic neurons (Treusch et al., 2011). Interestingly, mutations in the *C. elegans* human REST ortholog *spr-4*, which suppressed the *sel-12*/PSEN egg-laying defect (Lakowski et al., 2003), also enhanced the degeneration seen in the transgenic animals expressing Aβ in glutamatergic neurons (Lu et al., 2014). Modifying clathrin-mediated endocytosis in rat cortical neurons was similarly neuroprotective against Aβ aggregates (Treusch et al., 2011). In addition, early stage AD brains showed higher expression of REST target genes, while late stage AD and frontotemporal dementia (FTD) brains showed lower expression (Lu et al., 2014). Hence, REST may confer neuroprotective benefits in *C. elegans* and in humans (Lu et al., 2014).

The *C. elegans* Aβ model also proves useful in screens to identify drugs that disaggregate Aβ. The drug PBT2, an 8-hydroxy quinoline analog, reversed AD phenotypes in mice within days (Adlard et al., 2008). Similarly, *C. elegans* expressing inducible Aβ_1-42_, which become paralyzed within 48 h after induction, were protected against paralysis when exposed to PBT2 (McColl et al., 2012).

### *C. elegans lrp-1* FUNCTIONS SIMILARLY TO LRP2/MEGALIN, AN LDL RECEPTOR FAMILY MEMBER

In mammals, the LDL receptor family is responsible for many functions, including binding ligands for internalization and degradation and cholesterol metabolism (Brown and Goldstein, 1986; Mahley, 1988; Herz et al., 1992; Willnow et al., 1994). Binding of LRP1 to sAPP770 or full-length APP770, one of the APP isoforms (**Figure 1**), causes its internalization and degradation (Kounnas et al., 1995; Knauer et al., 1996); disrupting cell surface APP internalization with an LRP-antagonist increases sAPPα processing and full-length APP at the cell surface and decreases Aβ formation, suggesting that LRP1-APP interactions favor APP processing through the amyloidogenic pathway (Ulery et al., 2000). Apolipoprotein E and LRP2/megalin have also been implicated in Aβ clearance (Zlokovic et al., 1996; Deane et al., 2004; Carro et al., 2005).

*C. elegans* LRP-1 most closely resembles mammalian LRP2/megalin (Yochem and Greenwald, 1993; Yochem et al., 1999). *C. elegans* does not have the ability to synthesize cholesterol and, therefore, must rely on dietary sources (Hieb and Rothstein, 1968). Inactivation of *lrp-1* resulted in late larval lethality due to molting defects during the L3–L4 transition (Yochem et al., 1999). When wild-type *C. elegans* were grown in the absence of cholesterol, the molting defects of the *lrp-1* knockouts were phenocopied (Yochem et al., 1998; Wiese et al., 2012), suggesting that LRP-1 is involved in cholesterol uptake from the environment. LRP-1 is expressed in the epithelial hypodermal cells, hyp6 and hyp7, where it localizes to their apical surface (Yochem et al., 1999) and where *apl-1* is also expressed in adults (Niwa et al., 2008). Similarly, its mammalian counterpart, LRP2/megalin, is mainly expressed at the apical surface of epithelial cells (Cui et al., 1996; Morales et al., 1996; Willnow et al., 1996; Nielsen et al., 1998; Zheng et al., 1998; Hermo et al., 1999; Mizuta et al., 1999).

LRP2/megalin interacts with different domains of APP (Zlokovic et al., 1996; Pietrzik et al., 2004; Carro et al., 2005; Yoon et al., 2005; Cam and Bu, 2006). A physical interaction between APL-1 and LRP-1 has not been determined. Expression of LRP-1 with an N-terminal domain truncation did not rescue the lethality of *apl-1* null mutants, suggesting that sAPL-1 is not activating an *lrp-1* pathway (Hornsten et al., 2007). When *lrp-1* expression is decreased or when wild-type animals are deprived of dietary cholesterol, neurotransmission is affected (Wiese et al., 2012). However, *apl-1* null mutants die at an earlier stage in development than *lrp-1* null mutants, suggesting that *apl-1* functions in earlier developmental pathways that are necessary for survival.

## *C. elegans* AS A MODEL FOR OTHER NEURODEGENERATIVE DISEASES

### PTL-1 AS A TAU MODEL

**FIGURE 3:**
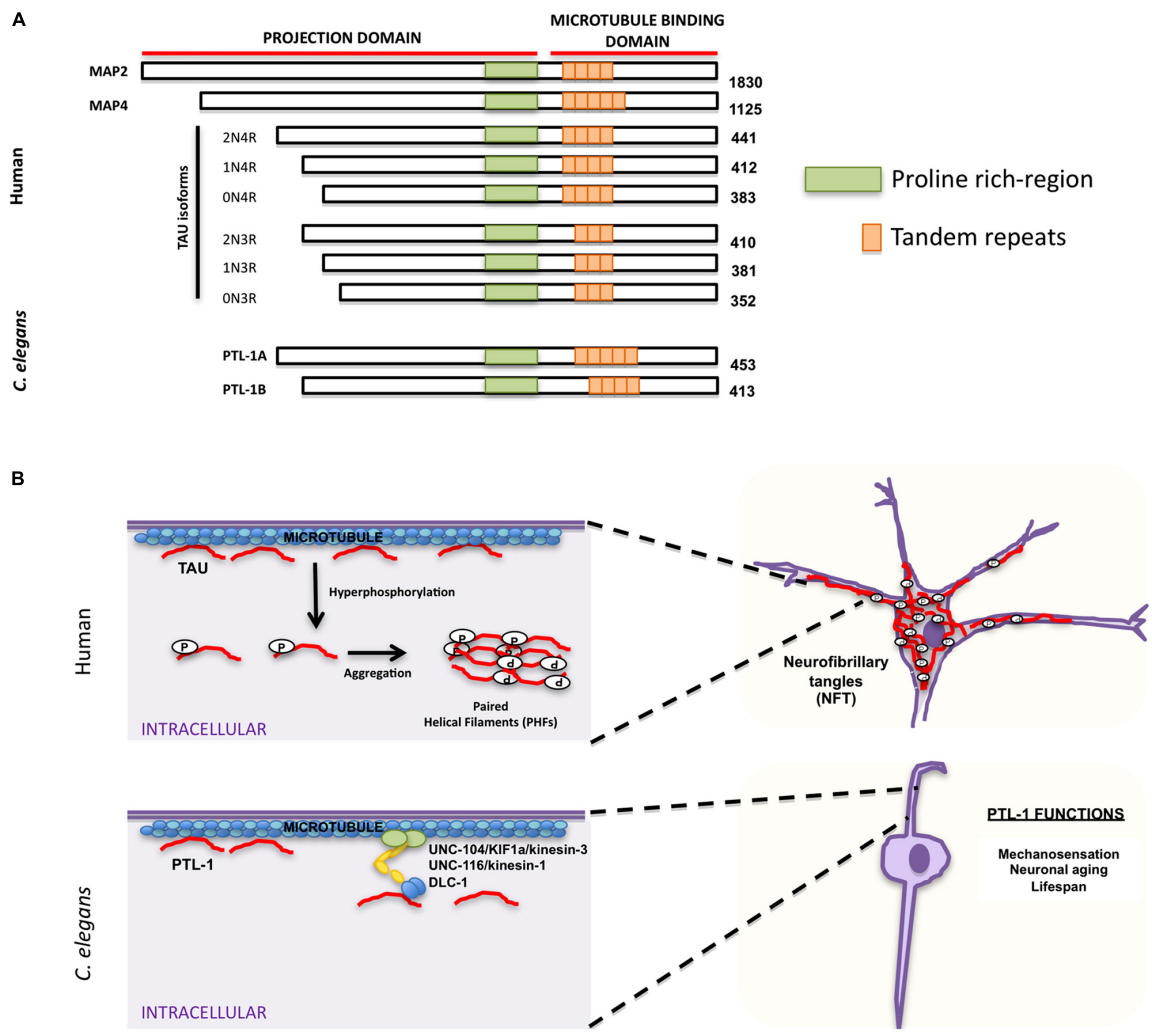
**Similarities and differences between human tau and *Caenorhabditis elegans* PTL-1. (A)** Schematic representation of human microtubule binding proteins (MAPs) family, including tau isoforms and *C. elegans* PTL-1 isoforms. **(B)** Comparison between tau functions in humans (top) and C. *elegans* PTL-1/tau functions (bottom). *Top*. Tau has a physiological role in promoting and maintaining microtubule stability. In pathological conditions tau is hyperphosphorylated and self-aggregates into paired helical filaments (PHFs) that can form intracellular neurofibrillary tangles (NFT). *Bottom. C. elegans* PTL-1/tau binds microtubules and induces microtubule assembly. It also affects synaptic transport through motor proteins UNC-104/KIF1a/kinesin-3, UNC-116/kinesin-1, and DLC-1/dynein. PTL-1/tau is also important for *C. elegans* mechanosensation and aging.

Accumulation of neurofibrillary tangles in cell bodies is another hallmark characteristic of AD and other neurodegenerative disorders. The major component of these tangles is tau, which belongs to the family of microtubule-associated proteins (MAPs) that includes MAP2 and MAP4 (Lee et al., 1988; Lewis et al., 1988; Goedert et al., 1989b; Chapin and Bulinski, 1991). MAPs share characteristic homology domains, including a proline-rich domain and a region of a variable number of tandem amino acid repeats (**Figure 3A**; Goedert et al., 1988, 1989a,b, 1992a; Lee et al., 1988; Lewis et al., 1988; Aizawa et al., 1990). Tau is the predominant MAP expressed in axons, while MAP2 is expressed in dendrites (Matus et al., 1981; Binder et al., 1985) and MAP4 is expressed in dividing cells (Bulinski and Borisy, 1980). MAPs bind microtubules and are responsible for promoting microtubule assembly and stability (reviewed in Amos and Schlieper, 2005). MAP family members appear to have redundant functions; mice in which tau was knocked out were viable, but showed increased levels of MAP1A (Harada et al., 1994), suggesting that upregulation of MAP1A can compensate for the lack of tau *in vivo*. Tau phosphorylation affects its ability to bind microtubules and can cause a conformational change that favors tubulin assembly (**Figure 3B**; Feijoo et al., 2005). Aberrant hyperphosphorylation of tau, however, impairs its ability to bind microtubules, thus resulting in their disassembly (Lindwall and Cole, 1984; Biernat et al., 1993; Bramblett et al., 1993). In addition, phosphorylated tau self-aggregates into PHFs and presumably generates the intracellular neurofibrillary tangles characteristic of AD patients (**Figure 3B**; Goedert et al., 1992b; Alonso et al., 1996, 1997, 2001; Billingsley and Kincaid, 1997).

Because of the functional redundancy of MAPs, their specific functions have been difficult to determine. *C. elegans* has only one tau homolog protein with tau-like repeats 1 (PTL-1; Goedert et al., 1996; McDermott et al., 1996). PTL-1 exists as two isoforms, PTL-1A and PTL-1B, with five or four tandem repeats, respectively (Goedert et al., 1996; **Figure 3A**). They have a high level of sequence homology with mammalian tau, especially in the C-terminal microtubule binding region (Goedert et al., 1996; McDermott et al., 1996). Both PTL-1A and PTL-1B bound microtubules *in vitro* and induced tubulin polymerization (Goedert et al., 1996; McDermott et al., 1996). PTL-1 is initially expressed embryonically in the epidermis of elongating embryos and in head neurons; in larval and adult animals PTL-1 is expressed mainly in the mechanosensory neurons mediating gentle body touch (**Figure 3B**; Goedert et al., 1996; Gordon et al., 2008), although transcriptional fusions show a wider expression pattern in neurons and stomatointestinal cells (Gordon et al., 2008).

Loss of *ptl-1*/tau results in an incompletely penetrant lethality at the same stage of embryogenesis (Gordon et al., 2008) at which PTL-1/tau expression is first observed (Goedert et al., 1996). *ptl-1*/tau mutants that escaped lethality showed normal development, but had a shortened lifespan, and although the overall integrity of microtubule structure appeared unaffected at the light microscopic level, there was a significant reduction in gentle touch sensitivity as compared to wild-type (Gordon et al., 2008; Chew et al., 2013). These touch defects were enhanced in mutants with defects in β- and α-tubulin, indicating that the absence of full-length PTL-1/tau disrupts mechanosensation, but it does so independently of tubulin (Gordon et al., 2008). In mutants in which only the C-terminal microtubule binding repeats of PTL-1/tau are deleted, touch sensitivity was identical to that of wild-type (Chew et al., 2013), suggesting that the N-terminal domain of PTL-1/tau is sufficient for gentle touch responses.

As *C. elegans* ages, mechanosensory touch neurons exhibit age-related morphological changes: cell bodies initially elaborate branches and axons subsequently display blebbing and branching (Pan et al., 2011; Tank et al., 2011; Toth et al., 2012). Strikingly, mechanosensory touch neurons in *ptl-1*/tau mutants displayed these aging characteristics at higher incidences and at an earlier stage than wild-type animals; GABAergic neurons also showed age-related phenotypes, such as ectopic branching (Chew et al., 2013). Expression of a human tau isoform (htau40) in *ptl-1*/tau mutants rescued the touch insensitivity, but not the morphological aging defects, indicating that htau40 shares some functional conservation with PTL-1/tau (Chew et al., 2013). Interestingly, when *ptl-1*/tau was expressed in Sf9 cells, cells projected neurite-like processes that were positive for PTL-1/tau immunoreactivity (Goedert et al., 1996) and that were indistinguishable from those visualized when htau40 was expressed in Sf9 cells (Knops et al., 1991; Chen et al., 1992). Although wild-type PTL-1/tau has not been reported to aggregate into fibrils, PTL-1/tau, like human tau, clearly has an essential role in maintaining neuronal integrity, controlling neuronal aging, and affecting lifespan (Goedert et al., 1996; Gordon et al., 2008). While there are no known mutations in tau that are associated with AD, tau mutations are associated with FTD with parkinsonism (FTPD-17), another form of dementia (Hutton et al., 1998; Poorkaj et al., 1998; Spillantini et al., 1998; see Section “Frontotemporal Dementia”).

### FRONTOTEMPORAL DEMENTIA

Frontotemporal dementia (FTD) is a group of neurodegenerative disorders characterized by severe brain frontotemporal lobar degeneration (reviewed in Rabinovici and Miller, 2010). In some cases it may be hard to distinguish between FTD and AD; however, FTD usually develops earlier in life and is more likely to have a genetic component (Lindau et al., 2000; Pasquier, 2005). Many mutations can cause FTD with or without motor neuron disease (Cruts et al., 2012). Two mutations have been well characterized and are associated with specific types of FTD: tau-positive FTD linked to chromosome 17 (FTD-17) and FTD caused by TDP43 proteinopathy (FTD-TDP43). Patients with FTD-17 suffer behavioral changes and often Parkinson-like motor problems. While mutations in the tau gene *MAPT* are not described in familial or sporadic AD, *MAPT* tau mutations are linked with FTD-17 (Hutton et al., 1998; Poorkaj et al., 1998; Spillantini et al., 1998).

**FIGURE 4:**
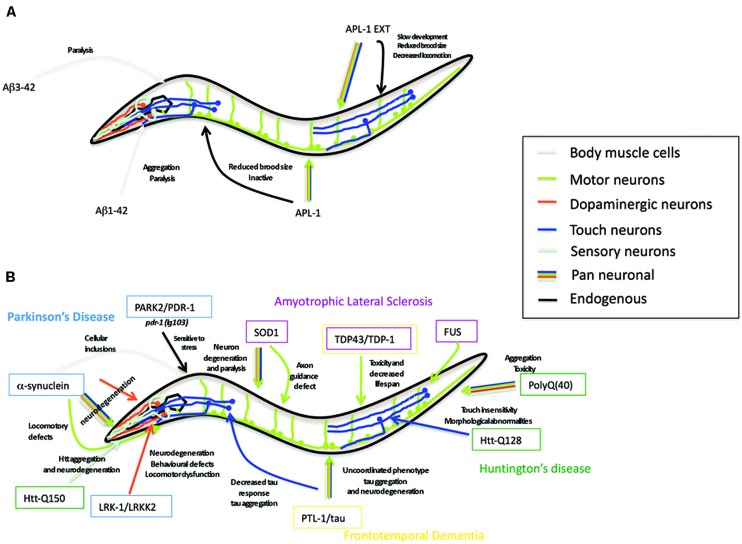
***Caenorhabditis elegans* as a transgenic model for AD and other neurodegenerative diseases. (A)** Summary of the Alzheimer’s disease models in *C. elegans* expressing human Aβ peptide or *C. elegans* full-length APL-1 or APL-1 extracellular domain (APL-1 EXT). Arrow color represents the tissues where transgenes were expressed. Phenotypes observed are next to the arrow. **(B)** Summary of the *C. elegans* models for Parkinson’s disease (PD), amyotrophic lateral sclerosis (ALS), frontotemporal dementia (FTD), and Huntington’s disease (HD). Genes modeling PD are shown in blue boxes, ALS in pink boxes, FTD in yellow boxes, and HD in green boxes. Arrow color represents tissues where transgenes were expressed. Phenotypes observed are written close to the arrow.

Several transgenic lines expressing human tau harboring FTD-17 mutations (htau-FTD-17) have been generated in *C. elegans* (**Figure 4B**) (see also Section “PTL-1 as a Tau Model”; Kraemer et al., 2003; Miyasaka et al., 2005; Brandt et al., 2009; Fatouros et al., 2012). Pan-neuronal transgene expression of wild type or htau-FTD-17 caused an uncoordinated phenotype that progressively worsened with age, an accumulation of insoluble tau, and neurodegeneration (Kraemer et al., 2003). Similarly, expression of htau-FTD-17 in touch neurons resulted in a decrease in the touch response due to neuritic abnormalities and tau accumulation (Miyasaka et al., 2005). Using these *C. elegans* models of tauopathy in forward genetic screens, Kraemer and co-workers identified two new factors, SUT-1 and SUT-2, that may participate in the pathological pathway activated by tau (Kraemer et al., 2003; Kraemer and Schellenberg, 2007; Guthrie et al., 2009). Moreover, down-regulation of the human SUT-2 homolog (MSUT-2) in mammalian cell lines caused a marked decrease in tau aggregation, suggesting that MSUT-2 may be a good candidate target for FTD therapies (Wheeler et al., 2010; Guthrie et al., 2011). More recently, Fatouros et al. (2012) generated two htau-FTD-17 transgenic models: one with a pro-aggregant mutated form of human tau (deletion of K280) and a second with mutated forms of human tau (I277P and I308P) that prevented tau aggregation. The tau (ΔK280) transgenic line had high levels of tau aggregation, which caused uncoordinated movement in adults, axonal defects, and alterations in presynaptic structures (Fatouros et al., 2012); the locomotory defects could be partially suppressed by a compound of the aminothioenopyridazine (ATPZ) class cmp16, suggesting that this compound may be neuroprotective (Fatouros et al., 2012). The tau (I277P and I308P) transgenic lines had low levels of tau aggregates and displayed only mild phenotypes with significantly less morphological abnormalities.

Accumulation of TDP-43 [transactive response (TAR) DNA-binding protein] is found in ∼50% of the cases of FTD (The Association for Frontotemporal Degeneration, 2014) and has numerous genetic causes. However, only one case has been reported with mutations in the TDP-43 gene (Borroni et al., 2009). *C.* e*legans* models overexpressing human TDP-43 or its *C. elegans* ortholog TDP-1 recapitulates some of the FTD phenotypes, including neurotoxicity and protein aggregation (see also Section “Amyotrophic Lateral Sclerosis”).

C9orf72 encodes a protein that regulates endosomal trafficking and autophagy in primary neurons and neuronal cells (Farg et al., 2014). It is expressed in multiple tissue types, including cerebellar cortex and spinal cord (DeJesus-Hernandez et al., 2011). Hexanucleotide (GGGGCC) repeat expansions in a non-coding region of C9orf72 are found in patients with amyotrophic lateral sclerosis (ALS) and FTD (DeJesus-Hernandez et al., 2011; Renton et al., 2011; Majounie et al., 2012), providing the first genetic link between the two diseases, although it remains unclear how C9orf72 hexanucleotide expansion triggers ALS and FTD pathology. Mutations in the *C. elegans* C9ORF72 ortholog *alfa-1* caused age-dependent motility defects, leading to paralysis and degeneration of GABAergic motoneurons (Therrien et al., 2013), suggesting that a loss-of-function mechanism is involved in the C9ORF72-dependent pathogenesis.

### PARKINSON’S DISEASE

Parkinson’s disease (PD) is a progressive neurodegenerative disorder that affects the control of body movements. The impaired motor control in PD is the result of the death of dopaminergic (DA) neurons (Hughes et al., 1992; Fahn and Sulzer, 2004). The disease is characterized by the accumulation of α-synuclein into neuronal inclusions called Lewy bodies (Lewy, 1912; Tretiakoff, 1919). Most PD cases are of unknown cause. However, ∼5–10% of PD cases are familial (Wood-Kaczmar et al., 2006) and include mutations in the following genes: α-synuclein, parkin (PRKN), leucine-rich repeat kinase 2 (LRRK2), PTEN-induced putative kinase 1 (PINK1), and ATP13A2 (Hardy, 2010).

α-synuclein is a presynaptic neuronal protein whose cellular function is not well understood, but may include controlling the supply of synaptic vesicles in neuronal terminals and regulating dopamine release. It is a small acidic protein (14 kDa) whose sequence can be divided into three domains: the N-terminal α-helical domain (amino acids 1–65), the central hydrophobic domain (residues 66–95), and the acidic carboxyl-terminal domain (residues 96–140; Recchia et al., 2004). Three mutations in the α-helical domain (A53T, A30P, E46K) are linked with autosomal dominant early onset PD, suggesting that these mutations can predispose to oligomer and fibril formation (Polymeropoulos et al., 1997; Krüger et al., 1998; Conway et al., 2000a,b,c; Zarranz et al., 2004). Because *C. elegans* has no α-synuclein ortholog, *C. elegans* models are based on transgenic worms overexpressing wild-type or mutant forms of human α-synuclein (**Figure 4B**). Although different transgenic lines showed some differences, most lines with pan-neuronal or DA neuronal expression of wild-type or mutated α-synuclein (A53T and/or A30P mutations) displayed locomotory defects and degeneration of dopamine neurons (Lakso et al., 2003; Kuwahara et al., 2006; Cao et al., 2010). Furthermore, downregulating the activity of the nuclease EndoG decreased α-synuclein toxicity in DA neurons (Büttner et al., 2013). EndoG is a mitochondria-specific endonuclease that mediates cellular death by apoptosis (Li et al., 2001). When cell death is induced, EndoG translocates from the mitochondria to the nucleus to fragment DNA (Li et al., 2001). Approximately 50% of the dopamine neurons expressing α-synuclein degenerate, whereas a mutation in *cps-6*, which encodes the *C. elegans* EndoG ortholog, rescues this degeneration (Büttner et al., 2013). Similar results were found in yeast, flies, and human cells suggesting that EndoG is a conserved requirement for α-synuclein toxicity (Büttner et al., 2013).

*C. elegans* models of α-synuclein overexpression-induced toxicity have also been examined by whole genome RNAi knockdown and microarray screenings (Vartiainen et al., 2006; Hamamichi et al., 2008; Kuwahara et al., 2008; van Ham et al., 2008). These screens highlighted the importance of endocytosis for ameliorating α-synuclein-dependent neurotoxicity (Kuwahara et al., 2008). Transgenic lines expressing α-synuclein specifically in body wall muscle cells produced inclusions as animals aged, resembling a feature of neurons in patients with PD; the number of inclusions decreased when genes affecting different biological processes, such as vesicle and lysosomal trafficking (W08D2.5/ATP13A2), lipid metabolism, and lifespan control (*sir-2.1*,*lagr-1*), were knocked down (van Ham et al., 2008). In a genome-wide microarray analysis to identify genes that were modulated in *C. elegans* overexpressing wild-type or A53T human α-synuclein, seven genes encoding components of the ubiquitin-proteasome machinery and 35 mitochondrial function genes were found to be upregulated, while nine genes encoding histones H1, H2B, and H4 were down regulated (Vartiainen et al., 2006). These data provide support for the role of the proteasome complex and mitochondrial proteins in mediating neurotoxicity.

Parkin (human PARK2) is a component of an ubiquitin E3 ligase that is part of the proteasome complex (Shimura et al., 2000). Mutations in PARK2 have been associated with early onset recessive forms of PD (Kitada et al., 1998; Poorkaj et al., 2004). *C. elegans* has a parkin ortholog, PDR-1. A truncated form of PDR-1(Δaa24–247) encoded by the in-frame deletion null allele *pdr-1*(*lg103*) had altered solubility and propensity to aggregate when expressed in cell lines, resembling parkin mutant proteins in PD (Springer et al., 2005). Furthermore, *pdr-1* mutants were hypersensitive to different proteotoxic stress conditions, suggesting that PDR-1/PARK2 mutations act to block the proteostasis machinery, thereby making it easier for proteins to abnormally fold and aggregate (Springer et al., 2005).

Mutations in LRRK2/leucine-rich repeat kinase 2 are the most common known cause of late-onset PD. LRRK2 belongs to the LRRK family; gain-of-function LRRK2 mutations interfere with chaperone-mediated autophagic functions and presumably decrease levels of α-synuclein degradation (Orenstein et al., 2013). Transgenic worms overexpressing pathogenic mutant forms of LRRK2 in DA neurons caused DA neurodegeneration (Liu et al., 2011; Yao et al., 2013). Interestingly, treatment of the transgenic worms with kinase inhibitors resulted in arrested neurodegeneration, suggesting that LRRK2 kinase activity is important for its pathogenesis (Liu et al., 2011; Yao et al., 2013).

Many studies have reported a link between toxin exposure and increased risk of PD. *C. elegans* has been used to test different toxins and help elucidate the mechanism by which they produce neurotoxicity. Administration of the 6-hydroxydopamine (6-OHDA) neurotoxin to *C. elegans* produced specific degeneration of dopamine neurons (Nass et al., 2002). By performing forward genetic and high-throughput chemical screens, mutations within the dopamine transporter *dat-1* were found to suppress 6-OHDA sensitivity (Nass et al., 2005) and bromocriptine, quinpirole, and acetaminophen, and plant extracts from *Bacopa monnieri* and *Uncaria tomentosa* were found to be neuroprotective (Marvanova and Nichols, 2007; Locke et al., 2008; Ruan et al., 2010; Jadiya et al., 2011; Shi et al., 2013). These data demonstrate that pathological characteristics of PD can be recapitulated in *C. elegans* models and used to investigate the mechanism by which α-synuclein and other PD proteins produce neurotoxicity and cause motor defects.

### AMYOTROPHIC LATERAL SCLEROSIS

Amyotrophic lateral sclerosis is a neurodegenerative disease characterized by the death of motor neurons in brain and spinal cord and progressive paralysis of the body (Hardiman et al., 2011). Approximately 10% of ALS cases are familial and associated with mutations in several genes. The most common mutation in familial ALS is found in the superoxide dismutase enzyme (SOD1) with more than 160 different mutations identified (Wroe et al., 2008). SOD1 is a ubiquitously expressed protein that converts the toxic radical superoxide anion to hydrogen peroxide. Although it is not clear yet how SOD1 mutations causes motor neuron degeneration, toxicity is likely generated by a gain-of-function mechanism (Valentine et al., 2005) and associated with misfolding and aggregation of the enzyme (Pasinelli and Brown, 2006).

Transgenic lines expressing mutant human SOD1 proteins have been successfully generated in *C. elegans* and recapitulate the motor neuron degeneration and paralysis characteristic of ALS patients (**Figure 4B**) (Witan et al., 2008; Gidalevitz et al., 2009; Wang et al., 2009; Li et al., 2014). The locomotion defect caused by pan-neuronal expression of the SOD1(G85R) mutant isoform was reduced when insulin signaling was decreased (Boccitto et al., 2012), suggesting that decreased insulin signaling increases the capacity of cells to prevent the accumulation of toxic non-soluble proteins and opening the possibility of finding new therapeutic targets. Similarly, when wild-type or mutant SOD1(G93A) was expressed exclusively in GABAergic motor neurons, animals showed an age-dependent paralysis and accumulation of wild-type and mutant SOD1(G93A), although defects were more severe in the mutant lines; interestingly, the SOD1 aggregates were soluble in the wild-type SOD1 lines and insoluble in the SOD1(G93A) lines (Li et al., 2014). In addition, motor neurons showed axonal guidance defects during development and caspase-independent cell death in adulthood in the wild-type and SOD1(G93A) lines (Li et al., 2014).

Other genes associated with ALS have also been modeled using *C. elegans*. TDP-43 [TAR DNA-binding protein] is a 43 kDa RNA binding protein identified as the main component of ubiquitinated protein aggregates (Tsuda et al., 2008; Murakami et al., 2012; Vaccaro et al., 2012a,b; Han et al., 2013; Therrien et al., 2013) found in patients with sporadic ALS (Neumann et al., 2006) and also in some cases of FTD (see Section “Frontotemporal Dementia”). TDP-43 is normally located in the nucleus of neurons, but dominant mutations in TDP-43 cause aberrant localization of TDP-43 in the cytoplasm, thereby preventing it from functioning in the nucleus (Gitcho et al., 2008; Kabashi et al., 2008; Sreedharan et al., 2008; Van Deerlin et al., 2008; Yokoseki et al., 2008; Ash et al., 2010). *C. elegans* has one TDP-43 ortholog, TDP-1. TDP-1 controls longevity and oxidative stress in the worm by regulating the insulin pathway (Vaccaro et al., 2012b). Overexpression of *tdp-1*/TDP-43 resulted in toxicity and decreased lifespan, analogous to the phenotypes found in ALS patients (Vaccaro et al., 2012b). In transgenic worms expressing TDP-43 harboring ALS-associated mutations, proteotoxicity affecting neuronal functions was induced. Similar results were found when the RNA binding protein FUS with ALS-related mutations was expressed in the nematode (Murakami et al., 2012; Vaccaro et al., 2012b).

Excess exposure to some pesticides and chemicals, such as the metalloid selenium, have been implicated in the etiology of ALS (Vinceti et al., 2009; Kamel et al., 2012; Malek et al., 2012). Exposure to high levels of sodium selenite in the worm induced neurodegeneration and resulted in paralysis (Estevez et al., 2012, 2014). When insulin pathway activity was reduced, the adverse effects of environmental selenium exposure was altered (Estevez et al., 2014). Overall, the *C. elegans* models have highlighted the possible importance of the insulin and autophagy pathways in the generation of ALS.

### HUNTINGTON’S DISEASE

Huntington’s disease (HD) is a progressive neurodegenerative disorder inherited through autosomal dominant mutations of the *IT15* gene. *ITI5* encodes the huntingtin protein, whose functions remain unknown (The Huntington’s Disease Collaborative Research Group, 1993). The mutations result in an N-terminal polyglutamine (polyQ) expansion (Goldberg et al., 1996; Mangiarini et al., 1996). In normal individuals, up to 34 repeats have been reported, whereas in HD aﬄicted individuals, up to 100 polyQ repeats have been recorded (The Huntington’s Disease Collaborative Research Group, 1993). The huntingtin-polyQ (HdhQ) proteins form aggregates, whose toxicity is determined by the length of the polyQ expansion and which cause swollen, disorganized, and ribosome-deficient endoplasmic reticulum and chromatin irregularities (Martindale et al., 1998). Eventually, cellular defects caused by the aggregates culminate in HD symptoms, which include involuntary movement, cognitive impairment, and loss of neurons in the striatum and deep layers of the frontal cortex (Martin and Gusella, 1986).

Although *C. elegans* does not have a huntingtin homolog, transgenic *C. elegans* models that express an N-terminal human huntingtin (htt) fragment with different numbers of CAG repeats have been used to model HD and identify genes that prevent polyQ aggregates (**Figure 4B**). The models generally express the repeats in specific neurons, such as the ASH sensory neurons, which are multi-modal sensory neurons that mediate avoidance to chemo- and mechanosensory stimuli. In transgenic animals expressing htt171 with 150 CAG repeats (htt171-Q150), 13% of the ASH neurons began to lose function after 8 days, suggesting an age-dependent degeneration (Faber et al., 1999). This loss of ASH function was reversed in a *ced-3/*caspase (Faber et al., 1999) or *hda-3/*HDAC (Bates et al., 2006) mutant background, suggesting that processes characteristic of apoptotic cell death and histone deacetylases play a role in HD (Dragunow et al., 1995). By contrast, the number of htt171-Q150 aggregates and neurodegeneration were enhanced when genes mediating autophagy, CREB, CREB binding proteins, and *pqe-1* were disrupted, suggesting that autophagy and activation of CREB target genes decreases htt171-Q150 aggregation and are neuroprotective (Faber et al., 2002; Bates et al., 2006; Jia et al., 2007). Transgenic animals expressing fewer CAG repeats (2, 23, and 95 polyQ) showed normal ASH function (Faber et al., 1999). The onset of behavioral defects are consistent with most cases of HD, in which symptoms usually appear during midlife (Vonsattel et al., 1985; Martin and Gusella, 1986; Strong et al., 1993; The Huntington’s Disease Collaborative Research Group, 1993; Gusella and MacDonald, 1995) and fewer than 10% of reported cases occur before the age of 21 (Farrer and Conneally, 1985; van Dijk et al., 1986; Nance, 1997; Siesling et al., 1997).

In a different HD model, htt57-Q128 was expressed in the touch mechanosensory neurons (Parker et al., 2001). These transgenic animals did not show neurodegeneration, but had a significantly reduced response to posterior touch and a milder defect in anterior touch response (Parker et al., 2001). The touch neurons contained polyQ aggregates and morphological abnormalities primarily along axonal processes (Parker et al., 2001). The touch insensitivity could be rescued by activating Sir2 sirtuins (Parker et al., 2005), which act through the DAF-16/FOXO transcription factor to promote longevity (Tissenbaum and Guarente, 2001). Similarly, in neuronal cell lines derived from knockin HdhQ111 mice, activation of sirtuins reduced the level of cell death (Parker et al., 2005). Additionally, in an RNAi based screen for genes that suppressed htt57-Q128 defects, identified *C. elegans* genes were also upregulated in the striatum of mouse HD models (Lejeune et al., 2012). Thus, *C. elegans* is a useful model to identify additional genes that may protect against or contribute to defects caused by polyQ expansions.

RNAi knockdown of *dnj-27*/ERdj5, an ER luminal protein upregulated in response to ER stress, exacerbated the impaired mobility observed when a Q40 transgene is expressed in body wall muscles, suggesting that *dnj-27* interacts with polyQ and protects against polyQ induced paralysis (Muñoz-Lobato et al., 2014).

*C. elegans* has the advantage that it is transparent, allowing visualization of the formation of that aggregates, including aggregates made by shorter polyQ tracts, whereas only longer tracts are visible in mammals (Brignull et al., 2006). To determine the threshold number of polyQ repeats needed to elicit a morphological and behavioral response, varying lengths of polyQ repeats were tested in *C. elegans*. Pan-neuronal expression of more than 40 polyQ led to variable protein aggregation and paralysis (Brignull et al., 2006). These data suggest that 40 polyQ may be the critical number of repeats to elicit HD symptoms and are consistent with unaffected humans who have up to 34 polyQ repeats and HD patients who have as few as 42 repeats (The Huntington’s Disease Collaborative Research Group, 1993).

Overall, *C. elegans* HD models illustrate that human huntingtin polyQs disrupt the morphology and function of sensory neurons. The genetic and RNAi screens highlight candidate genes that may be involved in HD pathogenesis in mammalian models and provide insights into genes that may serve a protective role against polyQ toxicity. In addition to HD, other diseases caused by polyQ repeats include spinocerebellar ataxias and spinal and bulbar muscular atrophy (Orr and Zoghbi, 2007). Hence, using *C. elegans* provides another approach toward determining how polyQ pathogenicity contributes to neurodegeneration.

## ADVANTAGES AND LIMITATIONS OF THE *C. elegans* MODEL

The use of *C. elegans* to study AD and other neurodegenerative diseases has, as many other models, many advantages as well as some drawbacks. Major advantages of *C. elegans* include the ability to perform forward genetic, RNAi, and high throughput chemical screens and the ease of generating transgenic lines. These benefits have been effective in informing the role of APP and the presenilins and identifying components of the γ-secretase complex. The function of APP and the pathways in which it acts are still unclear. *C. elegans* presents a complementary system to understand the function and pathways of an APP-related protein, APL-1. Furthermore, overexpression of APL-1 by mutation or by transgene induces phenotypes that converge on the insulin/DAF-16/FOXO pathways, similar to what has been found in mammals. Although APL-1 does not contain the Aβ sequence and *C. elegans* does not have β-secretase activity, transgenic lines that produce Aβ expression pan-neuronally or in muscle are being used to identify pathways that detoxify the Aβ aggregates, some of which also involve the insulin/DAF-16/FOXO pathways. Whether these models are relevant to human pathology or whether the pathways will be conserved in humans are unknown; however, they present alternative approaches to understanding neurodegenerative diseases for which there are currently few effective therapies. Human tau, as well as mutant tau isoforms, have also been expressed in the worm to recapitulate AD and FTD phenotypes. Recent findings have shown that PTL-1 regulates neuronal aging in the worm. These findings may be important to link aging and tau pathology in AD and FTD patients. Although *C. elegans* transgene models have many advantages, they also have several disadvantages. In *C. elegans*, transgenes are present as extrachromosomal arrays and are not integrated into the genome as they are in other systems; a few copies to several hundred copies of the transgene are present in the arrays, so the level of overexpression can be much higher than what is found *in vivo*. Fortunately, methods for single copy insertions have now been developed (Frøkjaer-Jensen et al., 2008).

AD is considered a multifactorial disease in which other risk factors, such as neuroinflammation, head trauma, and diabetes, may be important in the development of the disease. The *C. elegans* nervous system is simple compared to the human nervous system. This simplicity allows researchers to study neuronal function and neural circuits in a tractable system. However, the complex network of connections and cell interactions found in humans is not mimicked in *C. elegans* and this complexity may underlie some of the pathology of neurodegenerative diseases. Nevertheless, most of the pathways and signaling molecules in *C. elegans* are conserved between worms and mammals. The goal is to translate some of the *C. elegans* insights into understanding the pathology of AD and other neurodegenerative diseases and designing effective strategies to treat the diseases.

## Conflict of Interest Statement

The authors declare that the research was conducted in the absence of any commercial or financial relationships that could be construed as a potential conflict of interest.
